# DBCL-DFNet: Dual-Branch Contrastive Learning for Multi-Omics Dynamic Fusion

**DOI:** 10.3390/e28060616

**Published:** 2026-05-30

**Authors:** Yun Dang, Xiaoran Yan, Li Zhou, Dongxi Li

**Affiliations:** 1 College of Computer Science and Technology, Taiyuan University of Technology, Taiyuan 030024, China; 2023510419@link.tyut.edu.cn; 2 College of Artificial Intelligence, Taiyuan University of Technology, Taiyuan 030024, China; 3 College of Artificial Intelligence, Wuhan University, Wuhan 430072, China

**Keywords:** multi-omics integration, copula entropy, heterogeneous graph, GAT, mamba, contrastive learning

## Abstract

Multimodal omics data portray biological processes across molecular layers, yet their heterogeneity and high dimensionality hinder a unified representation. Existing integrative approaches either focus on local feature interactions or adopt static fusion, often overlooking the complementary global sequential context and the dynamic relevance among omics sources. Consequently, clinically critical tasks such as accurate cancer-subtype classification and therapy selection still lack sufficient accuracy and robustness. We introduce the Dual-Branch Contrastive Learning for Multi-Omics Dynamic Fusion Network (DBCL-DFNet), a dual-branch contrastive-learning framework that simultaneously encodes local heterogeneous graphs and global omics sequences, distills key features via contrastive objectives, and employs a dynamic attention mechanism for adaptive, data-driven fusion. Benchmarked on three public cancer multi-omics datasets, DBCL-DFNet outperforms both conventional machine-learning models and state-of-the-art deep-integration methods, establishing a competitive and reliable framework for multi-omics integration and demonstrating potential for precision-oncology decision-making. From an information-theoretic perspective, the framework integrates Copula-entropy-guided feature selection with mutual-information-maximizing contrastive alignment, providing a principled foundation for robust multi-omics integration.

## 1. Introduction

The rapid advancement of high-throughput sequencing technologies has generated an overwhelming amount of multi-omics data in life sciences, significantly enhancing our understanding of complex biological systems [1]. These technologies cover multiple dimensions, including genome-scale DNA sequencing, transcriptome mRNA expression profiling, epigenome DNA methylation detection, and small RNA (miRNA) expression analysis, among others [2]. Each type of omics data captures key aspects of biological processes from different perspectives, and their interconnections and interactions collectively drive disease progression and development [3]. The expanding dimensions of omics data provide a more comprehensive perspective for elucidating disease mechanisms, helping overcome the limitations of traditional genome-wide association studies (GWAS), such as identifying variants in non-coding sequences and revealing the value of important metabolic molecules in tumor progression [4,5]. Beyond increasing the dimensions of omics data, researchers are also committed to developing methods for integrating data from multiple omics layers. For example, integrating matched genomic and gene expression data enables the identification of genetic variants that influence gene expression levels across the genome, known as expression quantitative trait loci (eQTL) [6]. Fully leveraging these multi-omics data and analytical methods is an important direction for uncovering complex disease characteristics and advancing clinical applications.

Graph-based cancer subtype classification effectively captures complex relationships between samples that traditional methods often fail to utilize. By constructing patient similarity networks, graph models can reveal patient groups with similar molecular features, which is crucial for subtype identification [7]. Their visualization capabilities also help intuitively understand data structures and classification results [2].

Graph neural networks (GNNs) and graph attention networks (GATs) serve as core technologies for graph representation learning. Modeling omics data as a graph structure of biological entities and interactions allows GNNs to deeply explore intricate relationships within biological networks [8]. The attention mechanism introduced by GATs enables the model to learn the importance of different interactions and enhances its ability to focus on key biological linkages [7]. For example, the MOGAT framework integrates eight data types (mRNA, lncRNA, methylation, etc.) and improves cancer subtype prediction accuracy through a multi-head attention mechanism [8]; the AMOGEL model combines association rule mining (ARM) with GNNs to achieve better AUC scores than existing models in breast and kidney cancer data [6].

GNNs show great promise for multi-omics research, but there are still a number of obstacles to overcome. The first is the “curse of dimensionality–information loss” problem in high-dimensional data, which occurs when all original features are used directly to create a graph structure. This causes the graph size to increase exponentially and creates noise through erroneous correlations between redundant features. Conventional feature selection techniques may remove promising biomarkers and result in information loss even when they reduce dimensionality [9]. Moreover, current GAT variants mainly focus on direct interactions between nodes and fail to effectively integrate global information [6]. Second, homogeneous graphs may lead to a one-sided representation of feature associations: existing GAT models based on homogeneous graphs can only represent a single type of node or edge and cannot capture feature associations [10,11]. Third, static weights have insufficient adaptability for sample-level modality contributions: traditional multi-omics integration methods use fixed weights that cannot adapt to the varying importance of different modalities across samples [12,13].

From an information-theoretic perspective, the core challenge is to preserve discriminative information across heterogeneous omics while suppressing redundancy. To address this, we perform feature selection by combining the F3 score with Copula entropy. This hybrid strategy retains both class separability and information-theoretic relevance while reducing dimensionality. Furthermore, contrastive learning in our dual-branch encoder can be understood as maximizing a mutual-information lower bound between local graph views and global sequence views. Motivated by these insights, we design DBCL-DFNet as an information-aware multi-omics integration framework to overcome the above three limitations.

To address the above challenges, this paper employs heterogeneous graphs to model heterogeneous relationships, GAT+Mamba to capture local and global graph structures, a Transformer branch to preserve original sequence information, contrastive learning to align the dual branches, and dynamic attention to achieve sample-level adaptive fusion, thereby forming the Dual-Branch Contrastive Learning for Multi-Omics Dynamic Fusion Network (DBCL-DFNet) framework. The main contributions are as follows:

- We propose a multi-omics integration framework named DBCL-DFNet, which offers a robust and effective solution for cancer subtype classification based on multi-omics integration.
- The proposed model employs a dual-branch contrastive learning encoder to integrate local heterogeneous graphs with global sequences. This approach provides a unified perspective, effectively capturing critical features and their interrelationships, while simultaneously reducing graph complexity and preserving latent information.
- The model incorporates a dynamic attention mechanism to fuse the outputs from multiple omics encoders, thereby addressing the limitation of static weighting schemes and enhancing adaptability to sample-specific modality contributions.

## 2. Method

**Figure 1:**
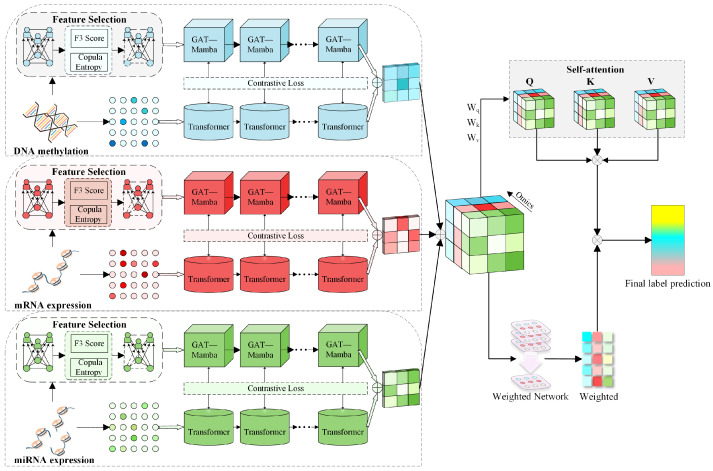
Schematic illustration of the Dual-Branch Contrastive Learning for Multi-Omics Dynamic Fusion Network (DBCL-DFNet), which integrates three types of omics data: DNA methylation, mRNA expression, and miRNA expression. The blue, red, and green branches represent DNA methylation, mRNA expression, and miRNA expression data, respectively.

The proposed multi-omics model for cancer subtype classification consists of three main modules, namely heterogeneous graph construction, the graph-sequence dual-branch structure, and the dynamic attention fusion mechanism. The overall workflow of our model is illustrated in Figure 1.

### 2.1. Heterogeneous Graph Construction

We construct a heterogeneous graph using carefully selected features to capture intricate patient-feature relationships while avoiding the computational overhead of a fully connected graph. Specifically, we create three complementary subgraphs: a patient similarity network, a feature similarity network, and a feature-patient network. These structures jointly reveal inter-patient affinities, intrinsic feature correlations, and associations between features and individual patients. The resulting heterogeneous graph encodes rich relational information that substantially enhances downstream node representation learning.

First, feature selection was conducted using the F3 score and Copula entropy to identify the most informative features from each omics modality [14,15]. The F3 score evaluates a feature’s discriminative power by measuring the degree of overlap between different classes, with higher scores indicating better separation. It is computed as follows:

(1) $$F3_{j}=\frac{2}{C(C-1)}\sum_{\left(e_{1},e_{2}\right)}\left(1-\frac{n_{\mathrm{overlap}}}{n_{\mathrm{total}}}\right)$$

where C is the number of classes, $n_{\mathrm{overlap}}$ is the number of overlapping samples between classes $e_{1}$ and $e_{2}$, and $n_{\mathrm{total}}$ is the total number of samples in these two classes. Copula entropy measures the dependence between a feature and the target variable, with lower values indicating higher dependence. It is defined as:

(2) $$\mathrm{CE}(x_{j},y)=\int_{0}^{1}\int_{0}^{1}c\left(u,v\right)\log c\left(u,v\right)\;du\;dv$$

where *u* and *v* are the uniform-transformed variables of the marginal distributions of $x_{j}$ and *y*, and c(u,v) is the corresponding Copula density function.

The F3 score and Copula entropy serve complementary roles in feature selection. The F3 score evaluates the discriminative power of a feature by measuring class separability based on sample overlap, which is intuitive and computationally efficient but primarily captures linear or rank-based separation. Copula entropy, in contrast, quantifies the statistical dependence between a feature and the target variable without assuming a specific functional form, thus capturing nonlinear and higher-order relationships that may be missed by the F3 score. By combining both measures, we retain features that are either well-separated in the original space or strongly dependent on the target in a potentially nonlinear manner. This hybrid strategy reduces dimensionality while minimizing the risk of discarding biologically relevant but nonlinearly associated features.

Subsequently, we constructed the heterogeneous graph to link the selected features and reveal their relationships. For each omics modality $X_{m}$, we computed the similarity matrix $S^{\left(m\right)}\in R^{N\times N}$ between patients using the Pearson correlation coefficient:

(3) $$S_{i,j}^{\left(m\right)}=\frac{\sum_{d=1}^{D_{m}}\left(x_{i,d}^{\left(m\right)}-{\bar{x}}_{i}^{\left(m\right)}\right)\left(x_{j,d}^{\left(m\right)}-{\bar{x}}_{j}^{\left(m\right)}\right)}{\sqrt{\sum_{d=1}^{D_{m}}{\left(x_{i,d}^{\left(m\right)}-{\bar{x}}_{i}^{\left(m\right)}\right)}^{2}}\sqrt{\sum_{d=1}^{D_{m}}{\left(x_{j,d}^{\left(m\right)}-{\bar{x}}_{j}^{\left(m\right)}\right)}^{2}}}$$

$D_{m}$ is the number of features in the *m*-th omics modality, $x_{i}^{\left(m\right)}\in R^{D_{m}}$ and $x_{j}^{\left(m\right)}\in R^{D_{m}}$ are the feature vectors of patients *i* and *j*, $x_{i,d}^{\left(m\right)}$ is the value of the *d*-th feature of patient *i* in the *m*-th omics modality, and ${\bar{x}}_{i}^{\left(m\right)}$ is the mean of patient *i*’s features in the *m*-th omics modality. According to the sparsity rate, we selected entries in the similarity matrix that exceeded a certain threshold to construct the sparse adjacency matrix $A_{m}$. Similarly, we constructed the feature similarity graph.

For the feature-patient association graph, we established connections between features and patients to represent the association between each feature and each patient. Specifically, for each feature $x_{i}$ and each patient $p_{j}$, we constructed an edge $\left(x_{i},p_{j}\right)$ to indicate that feature $x_{i}$ belongs to patient $p_{j}$.

By integrating feature selection and heterogeneous graph construction, our model effectively captures the most informative features and their relationships within each omics modality, providing a solid foundation for subsequent multi-omics data analysis.

### 2.2. Graph-Sequence Dual-Branch Structure

**Figure 2:**
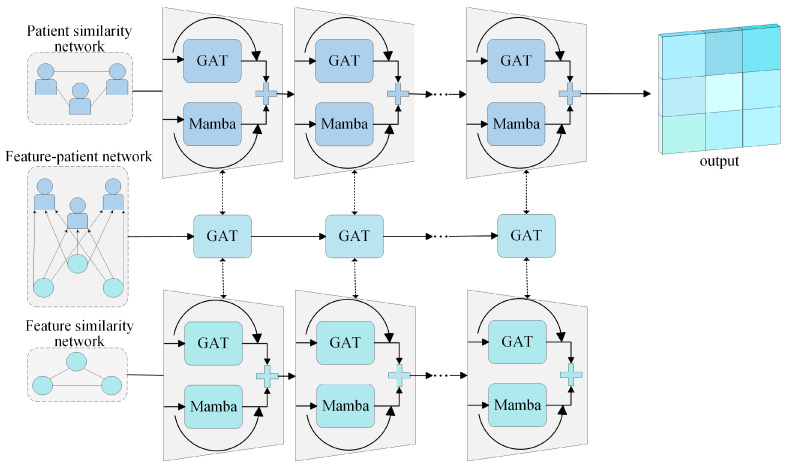
Workflow diagram of the heterogeneous graph branch using DNA methylation as an illustrative example. The three internal branches correspond to the patient similarity network, feature–patient association network, and feature similarity network within the heterogeneous graph. The blue and cyan modules are used to distinguish different representation-processing paths. Black arrows indicate the direction of information flow, curved arrows denote the interaction and residual fusion between GAT and Mamba modules, dotted arrows indicate cross-branch information transfer, and plus signs represent feature fusion.

In the graph branch, GAT dynamically assigns attention weights to the first-order neighbors of each node, thereby emphasizing local topological features. Meanwhile, Mamba arranges graph nodes into a sequential representation according to breadth-first traversal and leverages a selective state space model to capture long-range dependencies between distant node pairs, enabling effective modeling of global structural information [16,17]. The two modules extract complementary information from local and global perspectives, respectively, while avoiding functional redundancy. Their outputs are further fused through residual connections, allowing the resulting node representations to preserve both local contextual features and global structural dependencies. The detailed pipeline of the graph branch is illustrated in Figure 2.

Let *l* denote the layer index, and $Z_{m}^{\left(l-1\right)}\in R^{N\times D_{l-1}}$ be the node feature matrix output by the dual-branch encoder for modality *m* at layer l−1, where *N* is the number of nodes and $D_{l-1}$ is the feature dimension. The *j*-th row $Z_{m,j}^{\left(l-1\right)}$ corresponds to the features of node *j*. For the omics modality $X_{m}$, GAT learns the structural representation $G_{m}^{\left(l\right)}$ at layer *l* via the following attention-based convolution:

(4) $$G_{m}^{\left(l\right)}=\sigma \left(\sum_{j\in N(i)\cup \{i\}}\alpha_{ij}^{\left(l\right)}W_{m}^{\left(l-1\right)}Z_{m,j}^{\left(l-1\right)}\right)+Z_{m,i}^{\left(l-1\right)}$$

here, $W_{m}^{\left(l-1\right)}$ denotes the weight matrix of layer l−1. The term $\alpha_{ij}^{\left(l\right)}$ is the standard GAT attention coefficient computed by a shared attention mechanism. The input to this layer is given by $Z_{m}^{\left(l-1\right)}$. GAT learns the representation $G_{m}^{\left(l\right)}$ of layer *l* from $Z_{m}^{\left(l-1\right)}$ and $W_{m}^{\left(l-1\right)}$.

For the omics modality $X_{m}$, Mamba learns the structural representation of layer *l*, denoted ${M_{m}}^{\left(l\right)}$, through the following operations.

After batch-wise alignment and zero-padding, the node features are organized into a dense sequence $X^{\left(l-1\right)}\in R^{B\times T\times D}$, where *B* is the batch size, *T* is the maximum number of nodes in any single graph, and *D* is the feature dimension. An accompanying binary $\mathrm{mask}\in {\left\{0,1\right\}}^{B\times T}$ indicates valid nodes (1) and zero-padding (0). Leveraging the Structured State Space (S4) mechanism, Mamba models long-range dependencies within the sequence $X^{\left(l-1\right)}$. The core computation can be decomposed into the following steps.

Define the latent state $S_{t}\in R^{H}$ with state dimension *H*, initialized as $S_{0}=0$ (the zero vector). The state evolution follows discretized continuous-time state-space dynamics:

(5) $$S_{t}=AS_{t-1}+BX_{t}^{\left(l-1\right)}$$

here, $A\in R^{H\times H}$ is the state-transition matrix, and $B\in R^{H\times D}$ is the input projection matrix.

(6) $$O_{t}=CS_{t-1}+D_{\mathrm{ss}}X_{t}^{\left(l-1\right)}$$

Here, $C\in R^{D\times H}$ denotes the projection matrix from the hidden state to the output, and $D_{\mathrm{ss}}\in R^{D\times D}$ denotes the direct input-to-output mapping matrix (note that $D_{\mathrm{ss}}$ is distinct from the feature dimension *D*).

After aggregating the per-step outputs ${\left\{O_{t}\right\}}_{t=1}^{T}$ into the full-sequence encoding $Y_{m}^{\left(l\right)}={\left\{O_{t}\right\}}_{t=1}^{T}\in R^{B\times T\times D^{\prime }}$, we apply the mask to keep only the representations of valid nodes, perform dropout on the resulting ${\tilde{Y}}_{m}^{\left(l\right)}$, and finally add the processed vectors back to the input $M_{m}^{\left(l-1\right)}$ via a residual connection, producing the layer-*l* representation $M_{m}^{\left(l\right)}$:

(7) $$M_{m}^{\left(l\right)}=\mathrm{Dropout}\left({\tilde{Y}}_{m}^{\left(l\right)}\right)+Z_{m}^{\left(l-1\right)}$$

Finally, the final representation $Z_{m}^{\left(l\right)}$ is obtained by fusing the outputs of GAT and Mamba:

(8) $$Z_{m}^{\left(l\right)}=M_{m}^{\left(l\right)}+G_{m}^{\left(l\right)}$$

The input to the graph branch consists of heterogeneous graph nodes after feature selection, which reduces dimensionality but may also discard some global distribution information. To address this, we retain the original sequence data (without feature selection) and feed it separately into a Transformer branch [18]. This branch directly captures long-range dependencies among original features via multi-head self-attention, and its output complements that of the graph branch: the graph branch provides structured topological information, while the Transformer branch provides global patterns of the original sequence. The two branches are aligned via a contrastive loss, thereby fusing the two types of information.

For the omics modality $X_{m}$, the Transformer learns the *l*-th layer representation as follows.

The Transformer leverages multi-head self-attention to capture long-range dependencies among sequence elements, serving as the core component of feature processing. Given the *l*-th layer input $S_{m}^{\left(l-1\right)}\in R^{B\times T\times D_{l-1}}$, where *B* is the batch size, *T* is the sequence length, and $D_{l-1}$ is the feature dimension, learnable projection matrices are applied to generate queries $Q_{l}$, keys $K_{l}$, and values $V_{l}$.

We then compute the similarity between queries and keys, normalize it via Softmax to obtain attention weights, and simultaneously apply the ${\mathrm{mask}}^{attn}$ to suppress contributions from padded positions:

(9) $$A_{l}=\mathrm{Softmax}\left(\frac{Q_{l}K_{l}^{T}}{\sqrt{D_{l-1}}}\right)⊙{\mathrm{mask}}^{attn}$$

Next, the representation is split into multiple attention heads, the outputs from all heads are aggregated, and the original feature dimension is restored:

(10) $$\mathrm{MHSA}\left(S_{m}^{\left(l-1\right)}\right)=\mathrm{Concat}\left({\mathrm{head}}_{1},\ldots ,{\mathrm{head}}_{h}\right)W_{o}^{\left(l\right)}$$

where h denotes the number of attention heads and $W_{o}^{\left(l\right)}$ is the aggregation matrix. Here, ${\mathrm{head}}_{i}=\mathrm{Attention}\left(Q_{i},K_{i},V_{i}\right)$ with head-specific projections.

To preserve historical feature information and accommodate dimension changes across layers, residual connections along with projection operations are introduced:

(11) $$\mathrm{Proj}\left(S_{m}^{\left(l-1\right)}\right)=\left\{\begin{array}{cc} W_{res}^{\left(l\right)}S_{m}^{\left(l-1\right)}+b_{res}^{\left(l\right)}, & D_{l-1}\neq D_{l}\\ S_{m}^{\left(l-1\right)}, & D_{l-1}=D_{l} \end{array}\right.$$

where $D_{l}$ is the feature dimension of the *l*-th layer. When the dimensions differ between layers ($D_{l-1}\neq D_{l}$), a linear projection matrix $W_{res}^{\left(l\right)}\in R^{D_{l}\times D_{l-1}}$ together with bias $b_{res}^{\left(l\right)}\in R^{D_{l}}$ is applied to align the feature dimensions; otherwise, the features are passed through unchanged.

Subsequently, layer normalization is applied to the sum of the attention output and the residual connection, stabilizing training and accelerating convergence:

(12) $$S_{m}^{(l),\mathrm{norm}}=\mathrm{LN}\left(\mathrm{MHSA}\left(S_{m}^{\left(l-1\right)}\right)+\mathrm{Proj}\left(S_{m}^{\left(l-1\right)}\right)\right)$$

Ultimately, a two-layer feed-forward network enriches nonlinear feature expressiveness and distills higher-level semantics, yielding the final layer-*l* output $S_{m}^{\left(l\right)}$ of the Transformer.

For the loss function, we adopt a cooperative optimization strategy that jointly employs a classification loss and a contrastive loss [19]. Concretely, the adopted contrastive loss (InfoNCE) can be interpreted as maximizing a lower bound of the mutual information between the graph-branch representation and the Transformer-branch representation, thereby encouraging information-theoretic alignment across the two views.

Taking modality *m* as an example, the loss computation proceeds as follows.

The classification loss is used to supervise the model in learning discriminative features for category distinction. For modality *m*, the predicted logits $O_{m}^{\left(l\right)}\in R^{B\times C}$ (with *C* classes) are combined with the ground-truth labels $y\in R^{B}$ and the valid-sample $\mathrm{mask}\in {\left\{0,1\right\}}^{B}$ to compute the classification loss:

(13) $$L_{cls,m}^{\left(l\right)}=-\frac{1}{\sum_{i=1}^{B}{\mathrm{mask}}_{i}}\sum_{i=1}^{B}{\mathrm{mask}}_{i}\sum_{c=1}^{C}y_{i,c}\log(p_{i,c})$$

here, $\sum_{i=1}^{B}{\mathrm{mask}}_{i}$ represents the number of valid samples, $y_{i,c}\in \left\{0,1\right\}$ is the true label indicating whether the *i*-th sample belongs to class *c*, and $p_{i,c}$ is the predicted probability obtained by applying Softmax to the logits $O_{m}^{\left(l\right)}$.

The two sets of features for the contrastive loss come from two different processing paths: View 1 is the encoding result of the graph branch (GAT + Mamba) on the feature-selected heterogeneous graph nodes; View 2 is the encoding result of the Transformer branch on the original sequence data without feature selection. The contrastive loss forces the representations of these two views for the same sample to be as similar as possible, thereby enabling the model to fuse structured topological information with raw global sequence information and enhancing the discriminative power of the features.

For the *m*-th modality, given two sets of features $T_{m}^{\left(l\right)}\in R^{B\times T\times D_{l}}$ from the graph branch and $T_{m}^{\prime (l)}\in R^{B\times T\times D_{l}}$ from the Transformer branch, the process is as follows.

After performing $L_{2}$ normalization on the features, we obtain $T_{m}^{\mathrm{norm}}$ and $T_{m}^{\prime \mathrm{norm}}$. Then, the similarity matrix of the normalized features is computed:

(14) $${\mathrm{Sim}}_{i,j}=\frac{{\left(T_{m}^{\mathrm{norm}}\right)}_{i}\cdot {\left(T_{m}^{’\mathrm{norm}}\right)}_{j}^{T}}{\tau }$$

here, ${\left(T_{m}^{\mathrm{norm}}\right)}_{i}$ denotes the *i*-th sample feature vector of $T_{m}^{\mathrm{norm}}$, and ${\left(T_{m}^{\prime \mathrm{norm}}\right)}_{j}^{T}$ represents the transpose of the *j*-th sample feature vector of $T_{m}^{\prime \mathrm{norm}}$. τ is the temperature parameter. A ${\mathrm{mask}}^{attn}\in {\left\{0,1\right\}}^{B\times B}$ is introduced to mask out padded positions. The attention weights are then computed via the Softmax function:

(15) $$A_{m,i,j}=\frac{\exp\left({\mathrm{Sim}}_{i,j}\right)\times {\mathrm{mask}}_{i,j}^{attn}}{\sum_{k}\exp\left({\mathrm{Sim}}_{i,k}\right)\times {\mathrm{mask}}_{i,k}^{attn}},\;i,j\in \left[1,B\right]$$

where $A_{m,i,j}$ is the attention weight between sample *i* of the first view and sample *j* of the second view for modality *m*.

The contrastive loss is computed using cross-entropy, with the labels defined as the indices of the diagonal elements:

(16) $$L_{cont,m}^{\left(l\right)}=-\frac{1}{B}\sum_{i=1}^{B}\log\left(A_{m,i,i}\right)$$

The total loss is obtained by combining the classification loss and the contrastive loss, with $\lambda_{1}$ and $\lambda_{2}$ denoting the corresponding loss weights.

(17) $$L_{total,m}^{\left(l\right)}=\lambda_{1}L_{cls,m}^{\left(l\right)}+\lambda_{2}L_{cont,m}^{\left(l\right)}$$

### 2.3. Dynamic Attention Fusion Mechanism

Unlike static fusion strategies such as simple concatenation or fixed weighting, dynamic attention fusion generates modality-specific weights for each sample individually, thereby accommodating sample-specific differences in the contributions of different omics modalities [20,21]. Concretely, cross-modal multi-head attention is used to compute interactions among modality features, which are then passed through a two-layer fully connected network to produce sample-specific weight vectors. The weights are normalized via Softmax and used to adaptively weight the modality features. This allows the omics modality that is more discriminative for a given sample to obtain a higher fusion weight.

The fusion module learns the fused feature representation for classification prediction through the following operations. For the *m*-th omics input $\mathrm{input}1[m]\in R^{B\times D_{m}}$ and $\mathrm{input}2[m]\in R^{B\times D_{m}}$, where *B* denotes the batch size and $D_{m}$ represents the feature dimension of the *m*-th omics modality, the features are first concatenated and projected for enhancement. Subsequently, the enhanced features from all omics are stacked to form a sequence $\mathrm{features}\in R^{B\times M\times D_{\mathrm{hid}}}$, where *M* denotes the number of omics modalities and $D_{\mathrm{hid}}$ represents the dimension of the enhanced features.

Based on the multi-head attention mechanism, cross-modal interactions are performed on the multi-omics feature sequence. The query *Q*, key *K*, and value *V* are all defined as features. The multi-head attention is calculated as follows:

(18) $${\mathrm{head}}_{i}=\mathrm{Softmax}\left(\frac{Q_{i}K_{i}^{T}}{\sqrt{D_{\mathrm{hid}}/H}}\right)V_{i}$$

(19) $${\mathrm{attn}}_{\mathrm{output}}=\mathrm{Concat}\left({\mathrm{head}}_{1},...,{\mathrm{head}}_{H}\right)W_{o}$$

where *H* is the number of attention heads, $W_{o}\in R^{D_{\mathrm{hid}}\times D_{\mathrm{hid}}}$ is the aggregation matrix, and $\mathrm{attn}\_\mathrm{output}\in R^{B\times M\times D_{\mathrm{hid}}}$ denotes the attention output.

Based on the multimodal feature sequence, modality-specific weights are computed by a weight-generation network that first flattens the sequence into a vector representation, processes it with a two-layer feed-forward network, and finally applies a Softmax to yield ${\mathrm{weight}}_{\mathrm{dynamic}}\in R^{B\times M}$.

Finally, after obtaining $W_{\mathrm{output}}$ by weighting the attention output with ${\mathrm{weight}}_{\mathrm{dynamic}}$, $W_{\mathrm{output}}$ is passed through a two-layer feed-forward network that maps it into the classification space, producing the final classification logits $\in R^{B\times C}$, where *C* denotes the number of classes.

The idea of adapting to changing conditions rather than following a fixed schedule also appears in dynamic event-triggered control [22,23], which is conceptually related at a high level to our sample-specific dynamic weighting.

## 3. Experiments

### 3.1. Datasets

To validate our model, we applied it to three real-world cancer multi-omics datasets: the LGG dataset for classifying low-grade gliomas (Grade II and Grade III) [24], the RCC dataset for classifying renal cell carcinomas (KICH, KIRC, and KIRP) [25], and the BLCA dataset for classifying bladder urothelial carcinoma (low-grade vs. high-grade) [26]. The omics data were obtained from the TCGA cohort via the UCSC Xena platform [27]. Each dataset included DNA methylation, mRNA expression, and miRNA expression data, with matched samples only. Preprocessing involved handling missing values, normalizing features, and removing low-variance features. The details of the datasets are provided in Table 1.

### 3.2. Experimental Setup

To avoid information leakage, all data preprocessing steps, including Copula entropy and F3-score feature selection, as well as heterogeneous graph construction, were performed strictly within the training set of each fold; the test set did not participate in any preprocessing step. Specifically, we adopted stratified five-fold cross-validation: each dataset was randomly divided into five folds according to class proportions. In each round, one fold was used as the test set and the remaining four folds were used as the training set, resulting in five evaluation rounds. For each fold, feature selection and heterogeneous graph construction were first performed on the training set, and then the selected features and graph structure were mapped to the corresponding test set. All deep learning algorithms were implemented using the PyTorch 2.1.0 framework, and experiments were conducted on a Linux operating system with a vGPU-32GB GPU. The final results were reported as the mean ± standard deviation over the five test folds.

For the evaluation of binary classification results in cancer multi-omics data, we used Accuracy, AUROC, Recall, Precision, Specificity, NPV, and F1 score to assess the classification outcomes. For the evaluation of multi-class classification results in cancer multi-omics data, we used Accuracy, Macro F1, Micro F1, Weighted F1, Precision, and Recall to assess the classification outcomes.

### 3.3. Hyperparameter

To improve reproducibility, we summarized the key hyperparameter settings of DBCL-DFNet on the three datasets in Table 2. These hyperparameters include training-related parameters, branch-specific dropout rates, attention heads and layers, graph-construction parameters, and loss weights.

The feature sparsity rates for DNA methylation, mRNA expression, and miRNA expression were fixed at 0.9, 0.9, and 0.8, respectively, across all datasets. The random seed was fixed at 42 for reproducibility. For contrastive learning, the graph-branch representation and Transformer-branch representation of the same patient were treated as a positive pair, whereas representations from different patients within the same mini-batch were treated as negative pairs.

The hyperparameters in Table 2 were selected according to validation performance on the training folds. Dataset-specific hyperparameters, such as learning rate, dropout rate, GAT heads, and patient sparsity rate, were tuned because the three datasets differ in sample size, class distribution, and feature dimensionality. All hyperparameter selection was performed only on the training folds, and the test folds were not used for model selection or parameter tuning.

## 4. Results and Discussion

### 4.1. Evaluation of Multi-Omics Classification Performance

To effectively evaluate the classification accuracy of our model, we compared it with ten other multi-omics integration models. These included the K-Nearest Neighbors classifier (KNN) [28], Random Forest classifier (RF) [29], eXtreme Gradient Boosting (XGBoost) [25], Multi-Omics Graph Convolutional Network (MOGONET) [30], Graph Convolutional Network (GCN) [31], Cancer Molecular Subtype Diagnosis model (CancerSD) [32], Multi-Omics Dynamic Learning Integration Network (TMODINET) [33], Multi-Omics Hypergraph Integration Network (MORE) [34], Integrative Graph Convolution Networks (IGCN) [35], and SMODA [36].

The detailed comparison of classification results is shown in Table 3. As can be seen from the table, our model achieved the best performance on most evaluation metrics across the three datasets. For example, on the LGG dataset, our model achieved an accuracy approximately 3.2% higher than that of the second-best classification model (0.741 vs. 0.709). On the RCC dataset, our model was 1.4% more accurate than the second-best classification model (0.980 vs. 0.966). Interestingly, on the BLCA dataset, even with a smaller number of positive samples, our model still outperformed other machine learning and deep learning methods, with an accuracy 1.2% higher (0.976 vs. 0.964) and an F1 score 5.6% higher (0.868 vs. 0.812) than that of the second-best classification model. Overall, compared with the respective second-best methods, DBCL-DFNet improves accuracy by 3.2% (LGG), 1.4% (RCC), and 1.2% (BLCA), with an additional F1 gain of 5.6% on the highly imbalanced BLCA dataset. These results demonstrate that our model achieves competitive performance in both binary and multi-class classification tasks.

### 4.2. Ablation Study of Key Modules

To validate the effectiveness of each module, we conducted ablation experiments on three datasets using different variants of our proposed model. The settings are as follows: First, we directly constructed a patient similarity network and named this variant W/oHE. Second, to verify the effectiveness of the dual-branch structure, we performed the following operations: removing the Transformer branch (W/oTR), removing the GAT module from the graph branch (W/oGA), removing the Mamba module from the graph branch (W/oMA), and retaining only the Transformer branch (W/oGR). Third, we used only the original classification loss and named this variant W/oCL. Fourth, we replaced the dynamic attention mechanism with VCDN and named this variant W/oDA. Table 4 presents the classification results of the proposed model under different configurations.

The results show that the complete model (Ours) outperforms all variants across all datasets and metrics. Taking Accuracy on the LGG dataset as an example: the complete model achieves 0.741; removing only GAT (W/oGA) reduces it to 0.703, and removing only Mamba (W/oMA) reduces it to 0.697, with similar drops for both, indicating that GAT and Mamba each contribute complementary local and global information. Removing the entire graph branch (W/oGR) further reduces Accuracy to 0.674, and removing the Transformer branch (W/oTR) reduces it to 0.665, demonstrating that the graph branch and the sequence branch work synergistically and are both indispensable. After replacing the dynamic attention mechanism with VCDN (W/oDA), Accuracy on LGG, RCC, and BLCA drops to 0.669, 0.957, and 0.952, respectively, all lower than the complete model, confirming the effectiveness of sample-level dynamic weights. Removing the contrastive loss (W/oCL) also leads to performance degradation on all datasets (e.g., Accuracy on BLCA drops from 0.976 to 0.962), indicating that contrastive learning enhances the alignment of dual-branch features. The above ablation experiments not only verify the complementary strengths of each module but also provide empirical support for the overall effectiveness and robustness of the complete framework.

A closer look at Table 4 on the BLCA dataset reveals that W/oDA achieves the same Recall (0.720) as the full model but a lower F1 score (0.783 vs. 0.868). This implies that removing dynamic attention does not affect the identification of positive samples but leads to a drop in Precision (0.550 vs. 0.833), i.e., more false positives. The BLCA dataset is highly imbalanced (397 high-grade vs. 21 low-grade), which exacerbates over-prediction of the majority class. The dynamic attention mechanism adaptively reweights modality features per sample, helping to reduce false positives by focusing on discriminative cues. Without it, the model suffers from increased false positives and thus lower Precision and F1, while Recall stays unchanged.

### 4.3. Model Performance Across Different Omics Data Types

**Figure 3:**
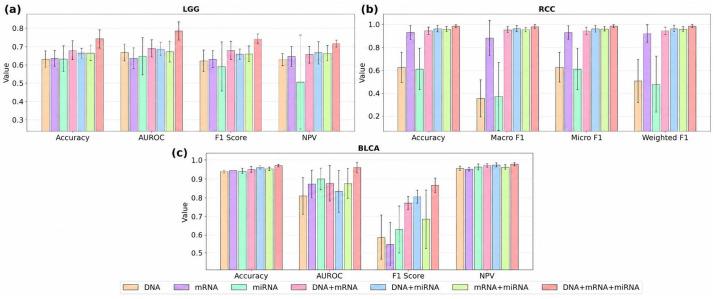
Performance comparison of the model across different modality combinations on three datasets (based on mean and standard deviation from 5-fold cross-validation). Subplots: (**a**) LGG dataset, (**b**) RCC dataset, (**c**) BLCA dataset.

To demonstrate the necessity of integrating multi-omics data, we conducted experiments using different modality combinations, including DNA, mRNA, miRNA, DNA + mRNA, DNA + miRNA, mRNA + miRNA, and DNA + mRNA + miRNA. The performance comparison under different settings is shown in Figure 3.

Clearly, the best results were achieved when DNA methylation, mRNA expression, and miRNA expression were simultaneously fed into the model, demonstrating that it effectively exploits cross-omics complementarity to capture biological signals inaccessible to any single modality. These results further confirm that integrating more comprehensive molecular information improves model performance and supports its potential utility in clinical research.

### 4.4. Interpretability Analysis Based on Dynamic Modality Weights

To further investigate the biological interpretability of DBCL-DFNet, we analyzed the dynamic attention weights learned by the multi-omics fusion module. Unlike static fusion strategies that assign fixed contributions to different omics modalities, the proposed dynamic attention mechanism generates sample-specific modality weights. Therefore, the learned weights can be regarded as modality-level indicators reflecting the relative contributions of DNA methylation, mRNA expression, and miRNA expression to the final prediction.

**Figure 4:**
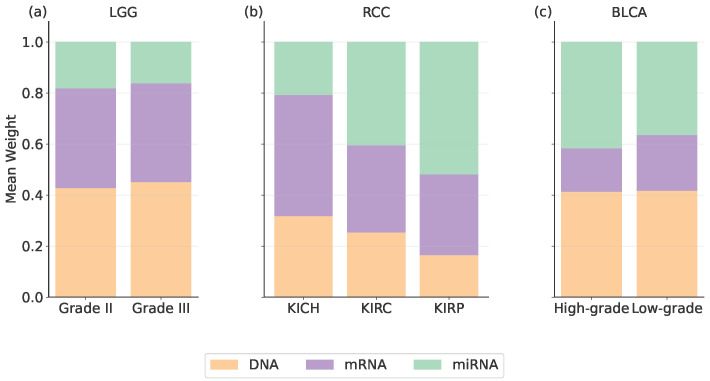
Average dynamic attention weights of DNA methylation, mRNA expression, and miRNA expression across different class labels. (**a**) LGG dataset (Grade II vs. Grade III). (**b**) RCC dataset (KICH, KIRC, KIRP). (**c**) BLCA dataset (high-grade vs. low-grade).

As shown in Figure 4, the learned dynamic weights reveal distinct modality-contribution patterns across different datasets and class labels. The values are reported in the order of DNA methylation, mRNA expression, and miRNA expression.

For the LGG dataset, the average weights are 0.428, 0.391, and 0.181 for Grade II samples, and 0.451, 0.387, and 0.162 for Grade III samples. These results indicate that both LGG grades assign higher weights to DNA methylation and mRNA expression than to miRNA expression.

For the RCC dataset, KICH shows the highest contribution from mRNA expression, with an average weight of 0.475. In contrast, KIRC and KIRP assign relatively higher weights to miRNA expression, with average weights of 0.405 and 0.519, respectively, suggesting subtype-specific modality preferences.

For the BLCA dataset, the average weights are 0.413, 0.171, and 0.416 for high-grade samples, and 0.417, 0.218, and 0.365 for low-grade samples. These results suggest that both BLCA grades rely more on DNA methylation and miRNA expression than on mRNA expression.

Overall, these observations indicate that DBCL-DFNet does not rely on fixed modality contributions but adaptively captures cancer- and subtype-specific molecular evidence. Therefore, the dynamic modality weights provide a quantitative modality-level explanation for the model’s classification behavior and offer useful clues for subsequent biological interpretation and precision-oncology research.

## 5. Conclusions

This study proposes the DBCL-DFNet framework to address core challenges in multi-omics data integration. The framework performs feature selection using the F3 score and Copula entropy, constructs a heterogeneous graph model integrating feature similarity, patient similarity, and feature-patient networks, and employs graph-sequence dual-branch learning with GAT-Mamba and Transformer to model both local and global features, aligning dual-branch representations via a contrastive loss. Additionally, it achieves adaptive multi-omics fusion through a dynamic attention mechanism. Experiments on three different cancer datasets demonstrate that the proposed methodology outperforms both conventional machine learning techniques and state-of-the-art deep learning-based multi-omics integration models. Quantitatively, compared with the best-performing state-of-the-art methods, DBCL-DFNet improves accuracy by 3.2% on LGG, 1.4% on RCC, and 1.2% on BLCA, and increases the F1 score by 5.6% on the highly imbalanced BLCA dataset.

Overall, DBCL-DFNet realizes effective integration of information-theoretic principles and deep multi-modal fusion, providing a reproducible and robust solution for cancer subtype classification. The framework establishes a systematic multi-omics data integration pipeline covering “feature selection–graph-sequence modeling–dynamic fusion”, enabling trustworthy and in-depth integration of multi-omics data. From a real-world application perspective, DBCL-DFNet can serve as a computational decision-support tool for precision oncology by assisting multi-omics-based cancer subtype classification, patient stratification, subtype-related risk assessment, and precision-oncology research. Rather than replacing pathological diagnosis or clinical judgment, it is expected to provide complementary molecular evidence for clinicians and biomedical researchers.

Although the proposed method achieves promising classification performance in cancer subtype identification, its generalization ability and fine-grained biological interpretability still have room for further improvement. In future work, we will further optimize the model structure and incorporate pathway-level enrichment analysis, biomarker discovery, and graph relationship interpretation to further explore the biological significance of the learned feature representations, thereby improving the clinical applicability and interpretability of the model.

## Figures and Tables

**Table 1:** Summary of multi-omics data.

No.	Dataset	Categories	Patients	Number of Features		
				DNA	mRNA	miRNA
1	LGG	Grade II: 254, Grade III: 268	522	8277	1166	287
2	RCC	KICH: 65, KIRC: 201, KIRP: 294	560	4107	2456	238
3	BLCA	High-grade: 397, Low-grade: 21	418	7999	2373	249

**Table 2:** Key hyperparameter settings of DBCL-DFNet.

No.	Hyperparameter	LGG	RCC	BLCA
1	Max epochs	200	100	200
2	Learning rate	$1.0\times 10^{-4}$	$1.0\times 10^{-3}$	$3.3\times 10^{-4}$
3	Weight decay	$4.0\times 10^{-5}$	$1.0\times 10^{-3}$	$1.6\times 10^{-6}$
4	GAT dropout	0.25	0.00	0.30
5	Transformer dropout	0.20	0.20	0.20
6	GAT/Transformer heads	4/4	2/4	5/4
7	GAT/Transformer layers	3/4	3/4	3/4
8	Selected graph features	200	200	200
9	Patient sparsity rate	0.88	0.80	0.90
10	Contrastive temperature τ	0.5	0.5	0.5
11	Loss weights $\left(\lambda_{1},\lambda_{2}\right)$	0.5, 0.5	0.5, 0.5	0.5, 0.5

**Table 3:** The classification performance is evaluated on three datasets.

Data	Metric	KNN	RF	XGBoost	GCN	MOGONET	CancerSD	TMODINET	MORE	IGCN	SMODA	Ours
**LGG**	Accuracy	0.667 ± 0.053	0.703 ± 0.056	0.678 ± 0.078	0.663 ± 0.044	0.674 ± 0.060	0.699 ± 0.029	0.691 ± 0.068	0.680 ± 0.042	0.692 ± 0.061	0.709 ± 0.042	**0.741 ± 0.049**
	AUROC	0.670 ± 0.052	0.704 ± 0.055	0.679 ± 0.078	0.704 ± 0.184	0.716 ± 0.050	0.765 ± 0.039	0.737 ± 0.046	0.734 ± 0.030	0.722 ± 0.075	0.765 ± 0.035	**0.783 ± 0.051**
	Precision	0.737 ± 0.066	0.735 ± 0.058	0.715 ± 0.108	0.712 ± 0.027	0.681 ± 0.057	0.713 ± 0.029	0.721 ± 0.094	0.698 ± 0.057	0.759 ± 0.103	0.756 ± 0.057	**0.787 ± 0.096**
	F1 Score	0.631 ± 0.058	0.694 ± 0.064	0.671 ± 0.088	0.659 ± 0.050	0.685 ± 0.056	0.699 ± 0.029	0.689 ± 0.071	0.677 ± 0.043	0.688 ± 0.061	0.707 ± 0.042	**0.739 ± 0.050**
	Recall	0.552 ± 0.116	0.664 ± 0.118	0.638 ± 0.107	0.575 ± 0.127	0.689 ± 0.102	0.694 ± 0.045	0.679 ± 0.046	0.683 ± 0.068	0.613 ± 0.080	0.646 ± 0.058	**0.709 ± 0.044**
	Specificity	**0.788 ± 0.077**	0.745 ± 0.083	0.721 ± 0.114	0.756 ± 0.055	0.657 ± 0.079	0.705 ± 0.038	0.704 ± 0.158	0.676 ± 0.121	0.775 ± 0.138	0.775 ± 0.070	0.775 ± 0.140
	NPV	0.630 ± 0.056	0.686 ± 0.071	0.657 ± 0.076	0.635 ± 0.063	0.674 ± 0.073	0.713 ± 0.029	0.704 ± 0.158	0.676 ± 0.121	0.654 ± 0.046	0.675 ± 0.041	**0.715 ± 0.018**
**RCC**	Accuracy	0.946 ± 0.028	0.950 ± 0.026	0.955 ± 0.022	0.952 ± 0.025	0.952 ± 0.025	0.964 ± 0.025	0.964 ± 0.018	0.954 ± 0.018	0.952 ± 0.028	0.966 ± 0.024	**0.980 ± 0.013**
	Macro F1	0.944 ± 0.025	0.950 ± 0.020	0.955 ± 0.019	0.951 ± 0.028	0.953 ± 0.022	0.962 ± 0.030	0.964 ± 0.026	0.953 ± 0.020	0.954 ± 0.029	0.963 ± 0.029	**0.977 ± 0.016**
	Micro F1	0.946 ± 0.028	0.950 ± 0.026	0.955 ± 0.022	0.952 ± 0.025	0.952 ± 0.025	0.964 ± 0.025	0.964 ± 0.018	0.954 ± 0.018	0.952 ± 0.028	0.966 ± 0.024	**0.980 ± 0.013**
	Weighted F1	0.947 ± 0.028	0.950 ± 0.026	0.955 ± 0.022	0.952 ± 0.024	0.952 ± 0.026	0.964 ± 0.024	0.964 ± 0.018	0.953 ± 0.018	0.952 ± 0.027	0.966 ± 0.024	**0.980 ± 0.013**
	Precision	0.949 ± 0.027	0.950 ± 0.025	0.955 ± 0.020	0.953 ± 0.023	0.956 ± 0.023	0.965 ± 0.023	0.961 ± 0.034	0.954 ± 0.021	0.953 ± 0.027	0.967 ± 0.023	**0.981 ± 0.013**
	Recall	0.946 ± 0.028	0.950 ± 0.026	0.955 ± 0.021	0.952 ± 0.025	0.952 ± 0.025	0.964 ± 0.025	0.968 ± 0.018	0.952 ± 0.023	0.952 ± 0.028	0.966 ± 0.024	**0.980 ± 0.013**
**BLCA**	Accuracy	0.955 ± 0.027	0.955 ± 0.022	0.964 ± 0.016	0.955 ± 0.014	0.948 ± 0.023	0.964 ± 0.012	0.962 ± 0.013	0.957 ± 0.012	0.959 ± 0.010	0.962 ± 0.014	**0.976 ± 0.007**
	AUROC	0.779 ± 0.191	0.684 ± 0.149	0.760 ± 0.175	0.920 ± 0.023	0.884 ± 0.160	0.962 ± 0.029	0.963 ± 0.018	0.932 ± 0.034	0.934 ± 0.067	0.964 ± 0.019	**0.966 ± 0.029**
	Precision	0.517 ± 0.329	0.600 ± 0.436	0.650 ± 0.369	0.513 ± 0.328	0.292 ± 0.407	0.650 ± 0.137	0.720 ± 0.205	0.383 ± 0.323	0.617 ± 0.100	0.639 ± 0.119	**0.833 ± 0.139**
	F1 Score	0.780 ± 0.191	0.680 ± 0.150	0.757 ± 0.178	0.685 ± 0.119	0.611 ± 0.168	0.791 ± 0.093	0.789 ± 0.073	0.671 ± 0.157	0.738 ± 0.081	0.812 ± 0.048	**0.868 ± 0.039**
	Recall	0.583 ± 0.382	0.383 ± 0.299	0.533 ± 0.356	0.340 ± 0.206	0.250 ± 0.335	0.570 ± 0.208	0.610 ± 0.223	0.350 ± 0.300	0.430 ± 0.186	0.670 ± 0.103	**0.720 ± 0.169**
	Specificity	0.975 ± 0.020	0.985 ± 0.020	0.987 ± 0.013	0.987 ± 0.014	0.985 ± 0.023	0.985 ± 0.006	0.980 ± 0.019	**0.990 ± 0.009**	0.987 ± 0.000	0.977 ± 0.015	**0.990 ± 0.009**
	NPV	0.978 ± 0.020	0.968 ± 0.016	0.976 ± 0.019	0.966 ± 0.011	0.961 ± 0.019	0.650 ± 0.137	0.980 ± 0.019	0.966 ± 0.017	0.970 ± 0.010	0.982 ± 0.006	**0.985 ± 0.009**

Bold values indicate the best performance, and underlined values indicate the second-best performance. For the binary-class datasets (LGG and BLCA), Precision and Recall are indicators for the high-grade (positive) class, while NPV and Specificity are indicators for the low-grade (negative) class. F1 Score is the macro-average (i.e., the average of the F1 scores of the positive and negative classes).

**Table 4:** Ablation studies are conducted on three datasets.

Data	Metric	W/oHE	W/oTR	W/oGA	W/oMA	W/oGR	W/oCL	W/oDA	Ours
**LGG**	Accuracy	0.697 ± 0.027	0.665 ± 0.013	0.703 ± 0.029	0.697 ± 0.039	0.674 ± 0.033	0.690 ± 0.039	0.669 ± 0.029	**0.741 ± 0.049**
	AUROC	0.748 ± 0.027	0.668 ± 0.031	0.765 ± 0.031	0.761 ± 0.039	0.742 ± 0.051	0.756 ± 0.030	0.743 ± 0.042	**0.783 ± 0.051**
	Precision	0.741 ± 0.046	0.690 ± 0.026	0.734 ± 0.052	0.761 ± 0.086	0.732 ± 0.086	0.725 ± 0.053	0.732 ± 0.063	**0.787 ± 0.096**
	F1 Score	0.696 ± 0.027	0.664 ± 0.013	0.701 ± 0.030	0.694 ± 0.038	0.668 ± 0.033	0.688 ± 0.039	0.665 ± 0.028	**0.739 ± 0.050**
	Recall	0.638 ± 0.033	0.634 ± 0.033	0.676 ± 0.070	0.623 ± 0.082	0.616 ± 0.125	0.646 ± 0.075	0.575 ± 0.065	**0.709 ± 0.044**
	Specificity	0.759 ± 0.067	0.697 ± 0.051	0.732 ± 0.095	**0.775 ± 0.117**	0.736 ± 0.143	0.736 ± 0.071	0.767 ± 0.090	0.775 ± 0.140
	NPV	0.665 ± 0.018	0.644 ± 0.012	0.684 ± 0.031	0.664 ± 0.034	0.653 ± 0.050	0.666 ± 0.041	0.632 ± 0.025	**0.715 ± 0.018**
**RCC**	Accuracy	0.970 ± 0.020	0.957 ± 0.017	0.964 ± 0.019	0.962 ± 0.026	0.952 ± 0.028	0.961 ± 0.027	0.957 ± 0.033	**0.980 ± 0.013**
	Macro F1	0.969 ± 0.018	0.955 ± 0.021	0.956 ± 0.029	0.958 ± 0.031	0.948 ± 0.034	0.955 ± 0.026	0.952 ± 0.033	**0.977 ± 0.016**
	Micro F1	0.970 ± 0.020	0.957 ± 0.017	0.964 ± 0.019	0.962 ± 0.026	0.952 ± 0.028	0.961 ± 0.027	0.957 ± 0.033	**0.980 ± 0.013**
	Weighted F1	0.970 ± 0.020	0.957 ± 0.018	0.964 ± 0.019	0.963 ± 0.026	0.952 ± 0.028	0.961 ± 0.027	0.957 ± 0.033	**0.980 ± 0.013**
	Precision	0.971 ± 0.020	0.958 ± 0.018	0.965 ± 0.018	0.965 ± 0.023	0.954 ± 0.026	0.962 ± 0.026	0.958 ± 0.032	**0.981 ± 0.013**
	Recall	0.970 ± 0.020	0.957 ± 0.017	0.964 ± 0.019	0.962 ± 0.026	0.952 ± 0.028	0.961 ± 0.027	0.957 ± 0.033	**0.980 ± 0.013**
**BLCA**	Accuracy	0.964 ± 0.011	0.954 ± 0.020	0.962 ± 0.009	0.959 ± 0.010	0.955 ± 0.018	0.962 ± 0.014	0.952 ± 0.014	**0.976 ± 0.007**
	AUROC	0.932 ± 0.057	0.888 ± 0.129	0.852 ± 0.162	0.926 ± 0.068	0.959 ± 0.027	0.780 ± 0.153	0.928 ± 0.064	**0.966 ± 0.029**
	Precision	0.700 ± 0.187	0.457 ± 0.293	0.717 ± 0.163	0.763 ± 0.225	0.594 ± 0.161	0.750 ± 0.247	0.550 ± 0.092	**0.833 ± 0.139**
	F1 Score	0.809 ± 0.044	0.741 ± 0.161	0.762 ± 0.060	0.738 ± 0.054	0.791 ± 0.049	0.750 ± 0.088	0.783 ± 0.040	**0.868 ± 0.039**
	Recall	0.620 ± 0.112	0.600 ± 0.424	0.480 ± 0.163	0.420 ± 0.144	0.670 ± 0.103	0.430 ± 0.186	**0.720 ± 0.243**	0.720 ± 0.169
	Specificity	0.982 ± 0.013	0.972 ± 0.016	0.987 ± 0.008	0.987 ± 0.014	0.970 ± 0.022	**0.990 ± 0.009**	0.967 ± 0.019	**0.990 ± 0.009**
	NPV	0.980 ± 0.006	0.980 ± 0.018	0.973 ± 0.009	0.970 ± 0.006	0.982 ± 0.006	0.970 ± 0.010	**0.985 ± 0.013**	0.985 ± 0.009

Bold values indicate the best performance, and underlined values indicate the second-best performance.

## Data Availability

DBCL-DFNet is available at https://github.com/dangyun943/DBCL-DFNet (accessed on 28 May 2026).

## References

[1] Baião A.R., Cai Z., Poulos R.C., Robinson P.J., Reddel R.R., Zhong Q., Vinga S., Gonçalves E. (2025). A technical review of multi-omics data integration methods: From classical statistical to deep generative approaches. Brief. Bioinform..

[2] Barylli M., Saha J., Buffart T.E., Koster J., Lenos K.J., Vermeulen L., Sheraton V.M. (2025). Biological Multi-Layer and Single Cell Network-Based Multiomics Models—A Review. arXiv.

[3] Hawkes G., Chundru K., Jackson L., Patel K.A., Murray A., Wood A.R., Wright C.F., Weedon M.N., Frayling T.M., Beaumont R.N. (2025). Whole-genome sequencing analysis identifies rare, large-effect noncoding variants and regulatory regions associated with circulating protein levels. Nat. Genet..

[4] Song T., Shi Y., Li Y., Hao D., Zhan K., Xu T., Chen R., He S. (2025). TOAnnoPriDB: An integrative database for trans-omic annotation and prioritization of non-coding variants across human genome. Sci. Bull..

[5] Strober B.J., Zhang M.J., Amariuta T., Rossen J., Price A.L. (2025). Fine-mapping causal tissues and genes at disease-associated loci. Nat. Genet..

[6] Tanvir R.B., Islam M.M., Sobhan M., Luo D., Mondal A.M. (2024). MOGAT: A Multi-Omics Integration Framework Using Graph Attention Networks for Cancer Subtype Prediction. Int. J. Mol. Sci..

[7] Tabakhi S., Vandermeulen C., Sudbery I., Lu H. (2024). Heterogeneous Graph Attention Network Improves Cancer Multiomics Integration. arXiv.

[8] Choi J.M., Chae H. (2023). moBRCA-net: A breast cancer subtype classification framework based on multi-omics attention neural networks. BMC Bioinform..

[9] Fang Z., Zhang X., Zhao A., Li X., Chen H., Li J. (2025). Recent Developments in GNNs for Drug Discovery. arXiv.

[10] Wang W., Chen H. (2023). Predicting miRNA-disease associations based on lncRNA–miRNA interactions and graph convolution networks. Brief. Bioinform..

[11] Li X., Ma J., Leng L., Han M., Li M., He F., Zhu Y. (2022). MoGCN: A Multi-Omics Integration Method Based on Graph Convolutional Network for Cancer Subtype Analysis. Front. Genet..

[12] Sammut S.J., Crispin-Ortuzar M., Chin S.-F., Provenzano E., Bardwell H.A., Ma W., Cope W., Dariush A., Dawson S.-J., Abraham J.E. (2022). Multi-omic machine learning predictor of breast cancer therapy response. Nature.

[13] Pan Y., Lei X., Zhang Y.C. (2022). Association predictions of genomics, proteinomics, transcriptomics, microbiome, metabolomics, pathomics, radiomics, drug, symptoms, environment factor, and disease networks: A comprehensive approach. Med. Res. Rev..

[14] Durante F., Sempi C. (2010). Copula Theory: An Introduction. Copula Theory and Its Applications.

[15] Güneş S., Polat K., Yosunkaya Ş. (2010). Multi-class f-score feature selection approach to classification of obstructive sleep apnea syndrome. Expert Syst. Appl..

[16] Veličković P., Cucurull G., Casanova A., Romero A., Liò P., Bengio Y. (2018). Graph Attention Networks. arXiv.

[17] Gu A., Dao T. (2023). Mamba: Linear-Time Sequence Modeling with Selective State Spaces. arXiv.

[18] Vaswani A., Shazeer N., Parmar N., Uszkoreit J., Jones L., Gomez A.N., Kaiser L., Polosukhin I. (2017). Attention is all you need. Proceedings of the Advances in Neural Information Processing Systems (NIPS 2017).

[19] van den Oord A., Li Y., Vinyals O. (2018). Representation Learning with Contrastive Predictive Coding. arXiv.

[20] Bahdanau D., Cho K., Bengio Y. (2014). Neural Machine Translation by Jointly Learning to Align and Translate. arXiv.

[21] Guo H., Jin X., Jiang Q., Wozniak M., Wang P., Yao S. (2024). DMF-Net: A Dual Remote Sensing Image Fusion Network Based on Multiscale Convolutional Dense Connectivity With Performance Measure. IEEE Trans. Instrum. Meas..

[22] Zhang D., Meng L., Liang L., Qin C., Liu D. (2026). Dynamic Event-Triggered Control for Human–Machine Cooperative Systems Based on Dynamic Authority Allocation. IEEE Trans. Syst. Man Cybern. Syst..

[23] Zhang D., Hao Y., Yuan Q., Qin C. (2026). Dynamic event-triggered approximate optimal consensus control for unknown nonlinear multi-agent systems via adaptive dynamic programming. ISA Trans..

[24] Network C.G.A.R. (2015). Comprehensive, Integrative Genomic Analysis of Diffuse Lower-Grade Gliomas. N. Engl. J. Med..

[25] Chen F., Zhang Y., Şenbabaoğlu Y., Ciriello G., Yang L., Reznik E., Shuch B., Micevic G., De Velasco G., Shinbrot E. (2016). Multilevel Genomics-Based Taxonomy of Renal Cell Carcinoma. Cell Rep..

[26] The Cancer Genome Atlas Research Network (2014). Comprehensive molecular characterization of urothelial bladder carcinoma. Nature.

[27] Goldman M.J., Craft B., Hastie M., Repečka K., McDade F., Kamath A., Banerjee A., Luo Y., Rogers D., Brooks A.N. (2020). Visualizing and interpreting cancer genomics data via the Xena platform. Nat. Biotechnol..

[28] Uddin S., Haque I., Lu H., Moni M.A., Gide E. (2022). Comparative performance analysis of K-nearest neighbour (KNN) algorithm and its different variants for disease prediction. Sci. Rep..

[29] Ho T.K. Random decision forests. Proceedings of the 3rd International Conference on Document Analysis and Recognition.

[30] Wang T., Shao W., Huang Z., Tang H., Zhang J., Ding Z., Huang K. (2021). MOGONET integrates multi-omics data using graph convolutional networks allowing patient classification and biomarker identification. Nat. Commun..

[31] Kipf T.N., Welling M. Semi-supervised classification with graph convolutional networks. Proceedings of the 5th International Conference on Learning Representations (ICLR).

[32] Bu Y., Liang J., Li Z., Wang J., Wang J., Yu G. (2024). Cancer molecular subtyping using limited multi-omics data with missingness. PLoS Comput. Biol..

[33] Du L., Gao P., Liu Z., Yin N., Wang X. (2024). TMODINET: A trustworthy multi-omics dynamic learning integration network for cancer diagnostic. Comput. Biol. Chem..

[34] Wang Y., Wang Z., Yu X., Wang X., Song J., Yu D.-J., Ge F. (2025). MORE: A multi-omics data-driven hypergraph integration network for biomedical data classification and biomarker identification. Brief. Bioinform..

[35] Ozdemir C., Vashishath Y., Bozdag S., Initiative A.D.N. (2025). IGCN: Integrative Graph Convolution Networks for Patient Level Insights and Biomarker Discovery in Multi-Omics Integration. Bioinformatics.

[36] Zhao J., Bao H., Guan P., Zhao X., Wang B., Yan Z., Zhao C., Zhao Y., Lu X., Xu G. (2026). SMODA: Interpretable Multimodal Omics Integration for Disease Classification and Subtype Discovery via Heterogeneous Transfer Learning. Anal. Chem..

